# A multiplex PCR for detection of knockdown resistance mutations, V1016G and F1534C, in pyrethroid-resistant *Aedes aegypti*

**DOI:** 10.1186/s13071-017-2416-x

**Published:** 2017-10-10

**Authors:** Jassada Saingamsook, Atiporn Saeung, Jintana Yanola, Nongkran Lumjuan, Catherine Walton, Pradya Somboon

**Affiliations:** 1 Graduate School, Department of Parasitology, Faculty of Medicine, Chiang Mai University, Chiang Mai, Thailand; 2 Department of Parasitology, Faculty of Medicine, Chiang Mai University, Chiang Mai, Thailand; 3 Department of Medical Technology, Faculty of Associated Medical Sciences, Chiang Mai University, Chiang Mai, Thailand; 4 Research Institute for Health Sciences, Chiang Mai University, Chiang Mai, Thailand; 5 School of Earth and Environmental Sciences, Faculty of Science and Engineering, University of Manchester, Manchester, UK

**Keywords:** *Aedes aegypti*, Insecticide resistance, *kdr*, Multiplex PCR

## Abstract

**Background:**

Mutation of the voltage-gated sodium channel (VGSC) gene, or knockdown resistance (*kdr*) gene, is an important resistance mechanism of the dengue vector *Aedes aegypti* mosquitoes against pyrethroids. In many countries in Asia, a valine to glycine substitution (V1016G) and a phenylalanine to cysteine substitution (F1534C) are common in *Ae. aegypti* populations. The G1016 and C1534 allele frequencies have been increasing in recent years, and hence there is a need to have a simple and inexpensive tool to monitor the alleles in large scale.

**Methods:**

A multiplex PCR to detect V1016G and F1534C mutations has been developed in the current study. This study utilized primers from previous studies for detecting the mutation at position 1016 and newly designed primers to detect variants at position 1534. The PCR conditions were validated and compared with DNA sequencing using known *kdr* mutant laboratory strains and field collected mosquitoes. The efficacy of this method was also compared with allele-specific PCR (AS-PCR).

**Results:**

The results of our multiplex PCR were in complete agreement with sequencing data and better than the AS-PCR. In addition, the efficiency of two non-toxic DNA staining dyes, Ultrapower™ and RedSafe™, were evaluated by comparing with ethidium bromide (EtBr) and the results were satisfactory.

**Conclusions:**

Our multiplex PCR method is highly reliable and useful for implementing vector surveillance in locations where the two alleles co-occur.

## Background

Insecticide resistance of *Aedes aegypti*, the primary mosquito vector of dengue, chikungunya, Zika and yellow fever viruses, is known to be widely spread throughout the world. There are two major resistance mechanisms, i.e. metabolic enzyme-based resistance and target site insensitivity [1]. Metabolic enzyme-based resistance is principally associated with three major enzyme groups: cytochrome P450 monooxygenases (P450s), esterases and glutathione S-transferases. Target site insensitivity in mosquitoes and other insects is associated with single or multiple mutations of the voltage-gated sodium channel (VGSC) protein, commonly referred to as knockdown resistance (*kdr*). Knockdown resistance is the important mechanism for resistance to pyrethroids and DDT [2]. Several mutations in VGSC of *Ae. aegypti* have been reported, but only a few of them have been confirmed to be associated with pyrethroid resistance. With reference to the homologous house fly VGSC sequence, a valine to glycine substitution at position 1016 within domain II of the VGSC (V1016G) is associated with resistance to type I and II pyrethroids, such as permethrin and deltamethrin, respectively [3, 4]. The V1016G mutation has been found in many countries in Asia, i.e. Indonesia [3, 5, 6], Thailand [7], Vietnam [8], Taiwan [9], Bhutan [10], Myanmar [11], Singapore [12], Malaysia [13] and China [14]. Moreover, a valine to isoleucine substitution in domain II (V1016I), conferring pyrethroid resistance, occurs amongst *Ae. aegypti* populations in Latin America [15–19], and also in Vietnam [20].

A second mutation, involving a phenylalanine to cysteine substitution at position 1534 within domain III (F1534C), is associated with resistance to type I pyrethroids [21]. The F1534C mutation has been reported from many countries in Asia [4–6, 8, 11–14, 22, 23] and Latin America [15–19]. Recently, it has also been reported from Africa [24].

Continuous and heavy use of space sprays in mosquito control programs are considered to be the cause of a dramatic increase in resistance and *kdr* allele frequencies [5, 19, 25]. The occurrence of *kdr* mutations in wild populations is expected to reduce the efficacy of *Ae. aegypti* mosquito control programs using pyrethroid insecticides [26, 27]. Therefore, monitoring the frequency of *kdr* alleles in the *Ae. aegypti* mosquito populations is important in the surveillance system of vector control programs.

Detection of *kdr* alleles in mosquitoes can be performed by several methods. Nucleotide sequencing is considered to be the most accurate method as a gold standard, but this method is expensive and not suitable for examining a large number of mosquitoes. A number of PCR-based techniques for detecting *kdr* alleles have been reported. An assay was optimized for use in a real time PCR machine, although the amplified products could also be detected via agarose gel electrophoresis [28]. An alternative technique using a heated oligonucleotide ligation assay (HOLA) was developed [7]. Although this assay does not involve the use of radioisotope or any specialized machine, it requires additional reagents that can contribute to increased costs. We recently developed allele-specific PCR-based assays (AS-PCR) to detect the F1534C [10] and V1016G mutations [4]. Although techniques are simpler and genotyping results can only be determined by gel electrophoresis, testing one sample requires two separate reactions, i.e. one for F1534C and the other V1016G. In addition, AS-PCR often shows a discrepancy with the DNA sequencing [10]. The purpose of this study was to develop a multiplex PCR to detect both F1534C and V1016G mutations in a single reaction. This technique can reduce the cost and time consumed in monitoring the mutant allele frequencies in many countries where both V1016G and F1534C mutations co-exist.

## Methods

### Mosquito samples

Five laboratory strains, two F1 hybrids, DNA samples and field-collected *Ae. aegypti* mosquitoes were used to develop, optimize and validate the multiplex PCR method. The three laboratory strains included a pyrethroid susceptible strain, PMD (homozygous wild type for both V1016 and F1534 alleles, V/V1016 + F/F1534), and two pyrethroid-resistant strains, PMD-R (homozygous wild type for the V1016 allele but homozygous mutant for the C1534 allele, V/V1016 + C/C1534) [21, 29], and UPK-R (homozygous mutant for G1016 allele but homozygous wild type for the F1534 allele, G/G1016 + F/F1534) [30]. The PMD and PMD-R strains originated from a rural area of Chiang Mai Province and have been maintained in our laboratory since 1997. The UPK-R strain was established from mosquitoes collected from Chiang Mai city and maintained since 2006. An F1 hybrid was derived from the cross between the PMD (male) and UPK-R (female) strains to produce heterozygous mutant for the G1016 allele but homozygous wild type for the F1534 allele, V/G1016 + F/F1534. Another F1 hybrid was derived from the cross between the PMD-R (male) and UPK-R (female) strains to produce heterozygous mutant for both G1016 and F1534 allele, V/G1016 + F/C1534.

The other two laboratory strains were Dagon Myothit North Yangon (YG) and Than Bya Zayet Monstate (MS) strains. These two strains originated from Myanmar, however, the *kdr* genotypes of each strain were not determined until the present study.

The DNA samples of *Ae. aegypti* collected from wild populations in Thailand, Myanmar, Cambodia, Bhutan and Pakistan were obtained from previous studies [4, 10].

Field-collected mosquito samples were obtained from larval surveys in various temples around Chiang Mai city, Thailand, as temples are numerous and readily accessible throughout Chiang Mai, and from households from several rural villages in Mae Taeng district. We also collected the immature stages from Myanmar (Yangon city) and Indonesia (Ternate Island and Soppeng Regency, South Sulawesi). The samples collected were reared to adulthood, identified morphologically and preserved in absolute ethanol until the multiplex PCR was performed.

### Development of multiplex PCR method

**Fig. 1:**
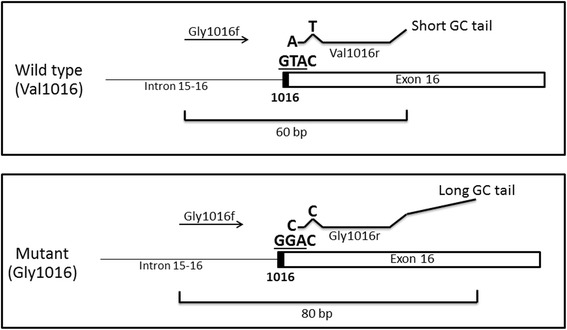
Schematic of the AS-PCR assay for detection of the V1016G mutation as described in Stenhouse et al. [4]

This assay was designed by combining two sets of primers. The first set was previously designed to genotype the V1016G mutation [28] and was used to develop an AS-PCR assay [4]. This set consists of three primers: a common forward primer (Gly1016f) and two specific reverse primers, Val1016r and Gly1016r, that differ in length and distinguish the V1016 and G1016 alleles, respectively (Fig. 1). In the case of a heterozygote, both products would be amplified.

**Fig. 2:**
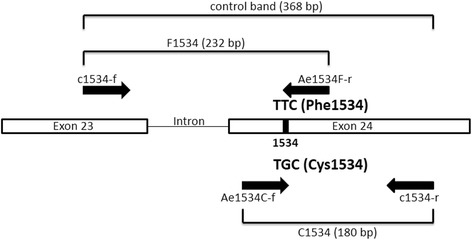
Schematic of the tetra primer AS-PCR assay developed herein for detection of the F1534C mutation

The second set of primers was newly designed using the web-based Primer3Plus software [31]. The cDNA nucleotide sequences of VGSC domains IIIS4-IVS2 of the *Ae. aegypti* PMD strain (GenBank: EU259810.1) and PMD-R strain (GenBank: EU259811.1), which were submitted by Yanola et al. [21], were used as reference sequences. This set of primers were designed to genotype the F1534C mutation (Fig. 2) and consisted of four primers (tetra primer PCR assay). In this assay, the outermost primers (c1534-f and c1534-r) amplified a control band of 368 bp. Two internal allele-specific primers, Ae1534F-r and Ae1534C-f, were designed to work in conjunction with the external primers to give amplified products of either 232 bp for the F1534 allele or 180 bp for the C1534 allele, respectively. In the case of a heterozygote, all three products would be amplified. All primer sequences used in this study are shown in Table 1.

**Table 1:** Sequences of primers used in this study

Primer name	Primer sequence (5′-3′)	Product size (bp)	Exon^a^
Direct sequencing			
IIP_F	GGTGGAACTTCACCGACTTC	581	15
IIS6_R	GGACGCAATCTGGCTTGTTA		16
Ge-IIIS6_F	GCTGTCGCACGAGATCATT	635	23
IIIS6_R	GTTGAACCCGATGAACAACA		25
Multiplex PCR			
1016 genotyping			
Gly1016f	ACCGACAAATTGTTTCCC		15–16^b^
Val1016r	[short GC tail]^c^ AGCAAGGCTAAGAAAAGGTTAATTA	60	16
Gly1016r	[long GC tail]^d^ AGCAAGGCTAAGAAAAGGTTAACTC	80	16
1534 genotyping			
c1534-f	GCGTACCTGTGTCTGTTCCA	368	23
c1534-r	GGCTTCTTCGAGCCCATCTT		24
Ae1534F-r	GCGTGAAGAACGACCCGA	232	24
Ae1534C-f	CCTCTACTTTGTGTTCTTCATCATCTG	180	24

^a^Exon from the *Ae. aegypti* VGSC gene. This transcript corresponds to VectorBase Transcript ID AAEL006019

^b^Intron between exon 15 and 16

^c^Short GC tail sequence: 5′-GCG GGC-3’

^d^Long GC tail sequence: 5′-GCG GGC AGG GCG GCG GGG GCG GGG CC-3′

For optimization, DNA samples extracted from the laboratory strains, PMD, PMD-R, UPK-R and (PMD-R × UPK-R) F1 hybrids were used. Our preliminary study revealed that the PCR conditions from previous reports [4, 28] were not suitable for our multiplex technique. Therefore, several PCR conditions were optimized for a total reaction volume of 10 μl: primer annealing temperature (50–65 °C), concentration of each primer (0.1–0.5 μM), *Taq* DNA polymerase concentration (0.05–0.5 unit), MgCl_2_ concentration (0.5–3.0 mM) and dNTP concentration (50–200 μM). Furthermore, the lowest amount of genomic DNA (DNA template) that still gave a clear result on agarose gel, i.e. the detection limit of this method, was also determined.

### V1016G and F1534C genotyping by multiplex PCR

Genomic DNA from each alcohol-preserved mosquito was extracted using DNAzol® reagent (Invitrogen, Carlsbad, California, USA). After PCR reactions, the amplified products were analyzed on 3% agarose gel with a low molecular weight DNA ladder (New England Biolab, Ipswich, Massachusetts, USA) used to estimate the band size. The electrophoresis was run for 50 min at 100 V in TBE buffer. The gel was then submerged in 0.5 μg/ml ethidium bromide (EtBr) (Invitrogen) solution for 15 min, de-stained for 5 min in distilled water, and visualized in a UV transilluminator.

Since EtBr is known to be a strong mutagen and is treated as hazardous waste, as alternatives we tried using Ultrapower™ (BioTeke, Beijing, China) and RedSafe™ (iNtRON Biotechnology, Gyeonggi-do, Korea) dyes, which are advertised as non-toxic and have no hazard waste. For Ultrapower™ staining, the dye solution (10,000×) was diluted 100-fold in 6× loading dye (New England Biolab), then 1 μl of diluted dye was mixed with 5 μl of PCR product. 1 μl of diluted dye was also added to 5 μl of the DNA ladder before loading on the gel. For RedSafe™ staining, 5 μl of this dye (20,000×) was mixed in with 100 ml molten agarose gel prior to gel pouring. Visualization was done immediately after gel electrophoresis.

### DNA sequencing

In order to validate the multiplex PCR method, the results of samples tested by this assay were compared by using DNA sequencing data obtained from previous studies [4, 10] as well as the present study. The IIS6 and IIIS6 regions of the VGSC gene, which encompass the V1016G and F1534C mutations, respectively, were amplified and purified. This method has been described previously [10]. For domain IIS6 amplification, each PCR was carried out in a 20 μl reaction volume, containing: 2 μl of DNA sample (50 ng), 0.4 units of Platinum *Taq* DNA polymerase (Invitrogen), 1.6 μl of 2.5 mM dNTPs mix (200 μM) (New England Biolabs), 0.6 μl of 50 mM MgCl_2_ (1.5 mM), 2 μl of 10× PCR buffer (1×) (Invitrogen), and 2 μl of 5 μM each of IIP-F (0.5 μM) and IIS6_R primers (0.5 μM) (Table 1), and made up to 20 μl with sterile water. The amplification consisted of 95 °C for a 2 min heat activation step, followed by 35 cycles of 95 °C for 30 s, 63 °C for 30 s and 72 °C for 30 s with a 2 min final extension step at 72 °C. Amplifying domain IIIS6 used the same conditions, but the primers were changed to Ge-IIIS6_F and IIIS6_R.

The amplified products were purified using Illustra™ ExoProStar™ 1-Step DNA purification reagent (GE Healthcare Life Sciences, Buckinghamshire, UK) and sent to Macrogen, Inc. (Seoul, Korea) for direct sequencing in both the forward and reverse directions. Sequence data were analyzed using Geneious software, version 5.3.6 (Biomatters Ltd., UK). Finally, for each mosquito sample, sequencing results were compared against the genotype previously obtained by the multiplex PCR method.

### Allele-specific PCR (AS-PCR)

The efficacy of multiplex PCR was compared with the AS-PCR methods for detecting F1534C and V1016G mutations as described in previous studies [4, 10]. The tested materials included DNA samples from the previous studies and newly extracted field samples.

## Results

### Development of multiplex PCR method

Tests for the optimization of PCR conditions resulted in the following multiplex PCR protocol. Each PCR reaction was performed in a 10 μl volume containing: 1 μl of DNA sample (25 ng), 0.4 units of Platinum *Taq* DNA polymerase (Invitrogen), 0.8 μl of 2.5 mM dNTPs mix (200 μM) (New England Biolabs), 0.3 μl of 50 mM MgCl_2_ (1.5 mM), 1 μl of 10× PCR buffer (1×) (Invitrogen), and primer concentrations: Gly1016f (0.5 μM), Val1016r (0.25 μM), Gly1016r (0.5 μM), c1534-f (0.25 μM), c1534-r (0.25 μM), Ae1534F-r (0.1 μM) and Ae1534C-f (0.5 μM), and made up to 10 μl with sterile water. The amplification consisted of 95 °C for a 2 min heat activation step, followed by 35 cycles of 95 °C for 30 s, 55 °C for 30 s and 72 °C for 30 s with a 2 min final extension step at 72 °C.

**Fig. 3:**
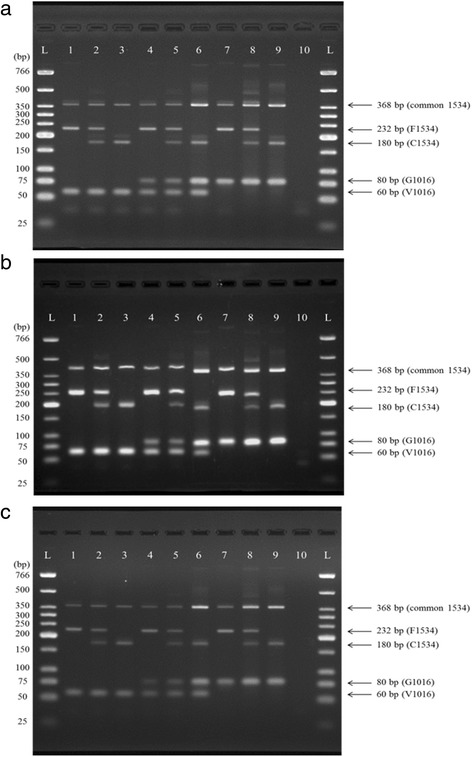
Gel electrophoresis results. **a** Stained with ethidium bromide. **b** Stained with Ultrapower™ dye. **c** Stained with RedSafe™ dye. All panels represent each of the nine possible genotypes. Lane L: contains low molecular weight DNA ladder. Lanes 1–9: contain PCR products by using a single mosquito DNA sample as template. Lane 1: VV/FF; Lane 2: VV/FC; Lane 3: VV/CC; Lane 4: VG/FF; Lane 5: VG/FC; Lane 6: VG/CC; Lane 7: GG/FF; Lane 8: GG/FC; Lane 9: GG/CC; Lane 10: negative control in which distilled water was used as the template in the PCR reaction

For these two polymorphic sites, there are nine possible genotypes, all of which were present in the laboratory and field-collected strains (Table 2, Fig. 3). The detection limit of this method was also evaluated by testing with a set of DNA dilutions of each genotype. The double heterozygous patterned sample (V/G1016 + F/C1534) needed the highest amount of DNA template (2 ng) to get a reliable result. Thus, the detection limit of this method was 2 ng of genomic DNA.

**Table 2:** Comparison of genotyping results for V1016G and F1534C mutations from multiplex PCR and DNA sequencing

Strain	Year of collection	Multiplex PCR genotyping/DNA sequencing (no. of samples)									
		VV/FF	VV/FC	VV/CC	VG/FF	VG/FC	VG/CC	GG/FF	GG/FC	GG/CC	Total
Laboratory strains											
PMD		10/10	0/0	0/0	0/0	0/0	0/0	0/0	0/0	0/0	10/10
PMD-R		0/0	0/0	10/10	0/0	0/0	0/0	0/0	0/0	0/0	10/10
UPK-R		0/0	0/0	0/0	0/0	0/0	0/0	10/10	0/0	0/0	10/10
(PMDxUPK-R) F1 hybrid		0/0	0/0	0/0	10/10	0/0	0/0	0/0	0/0	0/0	10/10
(PMD-RxUPK-R) F1 hybrid		0/0	0/0	0/0	0/0	10/10	0/0	0/0	0/0	0/0	10/10
YG		0/0	0/0	1/1	0/0	1/1	0/0	5/5	3/3	0/0	10/10
MS		0/0	0/0	0/0	0/0	2/2	2/2	0/0	5/5	1/1	10/10
Field-collected strains											
Thailand											
Chiang Mai city, Chiang Mai	2016	0/0	0/0	15/15	0/0	20/20	0/0	12/12	0/0	0/0	47/47
Mae Taeng district, Chiang Mai	2016	0/0	0/0	12/12	0/0	5/5	0/0	1/1	0/0	0/0	18/18
Mae Sariang district, Mae Hong Son^a^	2010	0/0	2/2	6/6	0/0	6/6	0/0	2/2	0/0	0/0	16/16
Total		10/10	2/2	44/44	10/10	44/44	2/2	30/30	8/8	1/1	151/151

^a^Mosquito DNA samples were obtained from Yanola et al. [10]

To compare the efficiency of three DNA staining dyes on this multiplex PCR, the PCR products from the same set of DNA templates were stained by EtBr (Fig. 3a), Ultrapower™ (Fig. 3b) and RedSafe™ (Fig. 3c), and the gels were visualized (with the best adjustment) under UV light. The EtBr and RedSafe™ staining methods gave clear and accurate results and the PCR product band sizes were correct, when determined by DNA Ladder. The RedSafe™ staining dye had a more faded result than EtBr; however, it had fewer non-specific bands. Ultrapower™ staining gave the brightest result, but bands were oversized when determined by DNA ladder. The C1534 (180 bp), F1534 (232 bp) and common 1534 (368 bp) bands were estimated as 200, 250 and 400 bp, respectively. The results were still interpretable by using these oversized bands, instead of original sizes.

### Comparison of DNA sequencing with the multiplex PCR method

A total of 151 samples from laboratory and field materials covering the nine genotypes were tested; all were successfully amplified by the multiplex PCR and sequenced. The sequences of all samples were in agreement with the multiplex PCR (Table 2). Thus, this multiplex PCR method has 100% specificity. However, there were limited numbers in a few genotypes (VV/FC, VG/CC and GG/CC) because they were rare in populations. The sequences of representative genotypes have been deposited in GenBank with accession numbers as follows: MF794972–3 (VV/FF), MF794974–5 (VV/FC), MF794976–7 (VV/CC), MF794978–9 (VG/FF), MF794980–1 (VG/FC), MF794982–3 (VG/CC), MF794984–5 (GG/FF), MF794986–7 (GG/FC) and MF794988–9 (GG/CC).

### Comparison of AS-PCR with the multiplex PCR method

A total of 169 field samples tested for F1534, C1534, V1016 and G1016 alleles by the AS-PCR were in agreement with the multiplex PCR, except one sample from Mae Sariang district which showed homozygous C/C1534 by AS-PCR, but heterozygous F/C1534 by the multiplex PCR (Table 3); the sequence of this sample (accession number MF794990) agrees with the multiplex PCR.

**Table 3:** Comparison of genotyping results for V1016G and F1534C mutations from multiplex PCR and AS-PCR

Strain	Year of collection	Total	Multiplex PCR genotyping/AS-PCR genotyping (No. of samples)					
			1534			1016		
			F/F	F/C	C/C	V/V	V/G	G/G
Thailand								
Mae Sariang district, Mae Hong Son^a^	2010	15	4/4	5/4	6/7^c^	9/9	2/2	4/4
Ranot district, Song Khla^a^	2008	10	0/0	3/3	7/7	10/10	0/0	0/0
Ubon Ratchathani city, Ubon Ratchathani^a^	2008	9	0/0	1/1	8/8	8/8	1/1	0/0
Myanmar								
Yangon city^a^	2008	10	4/4	6/6	0/0	10/10	0/0	0/0
Yangon city	2016	32	15/15	16/16	1/1	0/0	0/0	32/32
Cambodia								
Battambang city^a^	2008	10	0/0	0/0	10/10	10/10	0/0	0/0
Bhutan								
Phuntsholing city^a^	2006	12	12/12	0/0	0/0	0/0	0/0	12/12
Pakistan								
Lahore city^b^	2012	39	0/0	0/0	39/39	39/39	0/0	0/0
Indonesia								
Ternate Island	2017	20	19/19	1/1	0/0	0/0	1/1	19/19
Soppeng Regency, South Sulawesi	2017	12	12/12	0/0	0/0	0/0	0/0	12/12
Total		169	66/66	32/31	71/72	86/86	4/4	79/79

^a^Mosquito DNA samples were obtained from Yanola et al. [10]

^b^Mosquito DNA samples were obtained from Stenhouse et al. [4]

^c^One sample was homozygous C/C1534 by AS-PCR, but heterozygous F/C1534 by multiplex PCR and DNA sequencing

## Discussion

We have successfully developed the multiplex PCR method to detect both V1016G and F1534C *kdr* mutations in *Ae. aegypti* in a single reaction. The results of samples tested by our multiplex PCR method and DNA sequencing were in complete agreement to detect all nine possible *kdr* genotypic patterns derived from V1016G and F1534C mutations: VV/FF, VV/FC, VV/CC, VG/FF, VG/FC, VG/CC, GG/FF, GG/FC and GG/CC. According to previous studies, only three patterns, VV/CC, VG/FC and GG/FF, were found in Chiang Mai city [27] and several provinces throughout Thailand [4], which is in agreement with the current study. While VV/FC can be found in other provinces i.e. Mae Hong Son, Song Khla and Ubon Ratchathani, the wild type (VV/FF) and double homozygous mutant (GG/CC) were rarely detected, and only found in Myanmar. For VG/CC and GG/FC genotypes, these patterns have never been reported in Thailand, but have been found in Myanmar and elsewhere [5, 11]. Hence, we found these genotypic patterns in YG, MS and field-collected Yangon strains from Myanmar. The samples from Cambodia and Pakistan had only C1534 mutant allele (with VV/CC pattern), while Bhutan had only G1016 mutant allele (with GG/FF pattern).

In a previous study, we developed an AS-PCR assay to detect the F1534C mutation, but there were slight discrepancies between the AS-PCR results and those from sequencing [10]. A similar situation was found in the current study when we performed the AS-PCR. However, our multiplex PCR method showed no discrepancy with the sequence data; hence, this method is as good as DNA sequencing for both V1016G and F1534C mutations. Our multiplex PCR method is simple, has a lower cost and needs less special equipment compared to other molecular techniques, e.g. DNA sequencing, Taqman assay and Heated Oligonucleotide Ligation assay.

In this study, we used two alternative safe nucleic acid stains to compare with the traditional EtBr stain when determining the multiplex PCR results. EtBr has high sensitivity for DNA staining, provides accurate band sizes, and is cheaper than the safe dyes used. However, due to its carcinogenic property, it is preferable to use alternative safe stains, such as Ultrapower™ and RedSafe™, which are non-toxic, non-mutagenic, non-carcinogenic, and leave no hazardous waste. However, both have some disadvantages. Although the prestain safe dye Ultrapower™ staining had higher sensitivity than EtBr and RedSafe™, it gave oversized bands when determined by the DNA ladder. This may be because the dye bound to the double strand DNA PCR product before gel electrophoresis and thus reduced product mobility. This problem has been investigated with other prestain safe dyes, SYBR® Gold and SYBR® Green I [32]. Nonetheless, for this multiplex PCR method, the results were still interpretable by using these oversized bands, instead of true sizes. For RedSafe™ staining, which was mixed into the gel, the band sizes were accurate and had less non-specific bands than EtBr, but this had the lowest sensitivity. Following the manufacturer protocols, 1 μl RedSafe™ can stain 10 samples, while 1 μl Ultrapower™ can stain 100 samples. Because the price per volume is similar, Ultrapower™ is more cost effective by 10-fold. We therefore prefer to use Ultrapower™, particularly when testing a large number of samples.

Further development of multiplex PCR to include the serine to proline mutation (S989P) in domain II of VGSC is challenging, since P989 allele has a synergistic effect with G and C alleles in reducing the sensitivity of VGSC [30, 33]. However, adding an additional mutation would increase the number of possible genotypes and exponentially increase the complexity of banding patterns, potentially making the method unreliable due to difficulty in interpretation of the banding patterns. At present, detection of S989P may be less important in some countries where this mutation is known to co-occur or be highly associated with V1016G, such as Thailand, Singapore, Myanmar and China [4, 11, 12, 14]. However, in countries where P989 allele has not been detected or has a low frequency [5, 6, 9, 10, 13], monitoring this mutation in wild populations by an AS-PCR [14] may be a necessary component of the surveillance system.

## Conclusions

The multiplex PCR developed has high specificity and sensitivity to detect V1016G and F1534C *kdr* mutations in *Ae. aegypti* that allows all possible genotypes to be identified in a single step. This method was proved to be highly reliable and will be useful for monitoring mutant allele and genotype frequencies in wild populations throughout Thailand, and many other disease endemic countries in Asia, where these two alleles are prevalent.

## Abbreviations

AS-PCR: Allele-specific PCR
C1534: Cysteine allele at position 1534
DDT: Dichlorodiphenyltrichloroethane
EtBr: Ethidium bromide
F1: First generation of progeny
F1534: Phenylalanine allele at position 1534
F1534C: Phenylalanine to cysteine substitution at position 1534
G1016: Glycine allele at position 1016
GG/CC: G/G1016 + C/C1534
GG/FC: G/G1016 + F/C1534
GG/FF: G/G1016 + F/F1534
HOLA: Heated oligonucleotide ligation assay
IIIS4: Domain III segment 4 of VGSC gene
IIIS6: Domain III segment 6 of VGSC gene
IIS6: Domain II segment 6 of VGSC gene
IVS2: Domain IV segment 2 of VGSC gene
*kdr*: Knockdown resistance
MS: Than Bya Zayet Monstate strain
P450s: Cytochrome P450 monooxygenases
S989P: Serine to proline substitution at position 989
TBE: Tris-Borate-EDTA buffer
V1016: Valine allele at position 1016
V1016G: Valine to glycine substitution at position 1016
V1016I: Valine to isoleucine substitution at position 1016
VG/CC: V/G1016 + C/C1534
VG/FC: V/G1016 + F/C1534
VG/FF: V/G1016 + F/F1534
VGSC: Voltage-gated sodium channel
VV/CC: V/V1016 + C/C1534
VV/FC: V/V1016 + F/C1534
VV/FF: V/V1016 + F/F1534
YG: Dagon Myothit North Yangon strain
